# Physiological Biomarkers Assessed by Low-Tech Exercise Tests Predict Complications and Overall Survival in Patients Undergoing Pneumonectomy Due to Lung Cancer

**DOI:** 10.3390/cancers13040735

**Published:** 2021-02-10

**Authors:** Tomasz Marjanski, Damian Wnuk, Robert Dziedzic, Marcin Ostrowski, Wioletta Sawicka, Ewa Marjanska, Witold Rzyman

**Affiliations:** 1 Thoracic Surgery Department, Medical University of Gdansk, Sklodowskiej-Curie 3A, 80-211 Gdansk, Poland; dziedzic@gumed.edu.pl (R.D.); marcin.ostrowski@gumed.edu.pl (M.O.); wrzyman@gumed.edu.pl (W.R.); 2 Department of Physical Therapy, Medical University of Gdansk, Sklodowskiej-Curie 3A, 80-211 Gdansk, Poland; damian.wnuk@gumed.edu.pl; 3 Department of Anaesthesiology and Intensive Care, Medical University of Gdansk, Sklodowskiej-Curie 3A, 80-211 Gdansk, Poland; wioletta.sawicka@gumed.edu.pl; 4 Faculty of Management and Economics, Gdansk University of Technology, Narutowicza 11/12, 80-233 Gdansk, Poland; ewa.marjanska@zie.pg.edu.pl

**Keywords:** lung cancer, pneumonectomy, complications, overall survival, 6-min walking test

## Abstract

**Simple Summary:**

Preoperative results of the 6-min walking test help to identify risk of postoperative complications and increased mortality in patients undergoing lobectomy for lung cancer. The aim of the study was to validate the value of 500 m in 6MWT as an indicator, which differentiates risk of complications in patients undergoing pneumonectomy. 125 patients who underwent pneumonectomy at Thoracic Surgery Department between 2009 and 2018. Additionally, on the day before the surgery, patients performed the 6-min walking test. We analyzed 93 men and 32 women with a median age of 63 years. The cut-off value of 500 m identified patients with increased 90-day mortality, first-year mortality, and overall survival. Patients who covered a distance ≤ 500 m had an increased risk of atrial fibrillation and cardiac complications. Patients who do not reach the distance of 500 m in 6-min walking test have a high risk of early postoperative death after pneumonectomy.

**Abstract:**

Due to its debilitating character pneumonectomy this is last-resort procedure. Preoperative results of the 6-min walking test (6MWT) help to identify high risk of postoperative complications and increased mortality in patients undergoing lobectomy for lung cancer. The aim of the study was to validate the value of 500 m in 6MWT as an indicator, which differentiates risk of complications in patients undergoing pneumonectomy. 125 patients who underwent pneumonectomy at Thoracic Surgery Department between 2009 and 2018. On the day preceding the surgery, patients underwent 6MWT. The patients were in median age of 63 years. The cut-off value of 500 m identified patients with increased 90-day mortality [17.9% vs. 3.5%, odds ratio (OR) 6.271, 95% confidence interval (CI) 1.528–25.739], first-year mortality (30.7% vs. 11.6%, OR 3.378, 95% CI 1.310–8.709), and overall survival (*p* = 0.02). Patients who covered a distance ≤ 500 m had an increased risk of atrial fibrillation (35.9% vs. 16.3%, OR 2.880, 95% CI 1.207–6.870) and cardiac complications (38.4% vs. 19.8%, OR 2.537, 95% CI 1.100–5.849). Patients unable to reach 500 m in 6MWT are in a high risk of postoperative death after pneumonectomy, what may be a result of increased frequency of postoperative cardiac complications. Poor result of 6MWT is a predictor of worse overall survival.

## 1. Introduction

Recent advances in early detection and screening of lung cancer decrease the number of patients with advanced diseases requiring radical surgery [1,2]. However, 10% of patients referred for resection still require pneumonectomy [3]. This number is decreasing due to earlier diagnosis and advances in bronchial and vascular sleeve resections [4]. The decision to perform pneumonectomy must balance the oncological benefits with the debilitating character of this procedure. The consequences of pneumonectomy go way beyond the prompt reduction of the pulmonary parenchyma volume. Overload of circulatory system and vulnerability to pulmonary infections reduce the overall survival. The prognosis related to disease-free survival at every stage of lung cancer is well-documented [5]. However, competing risks of death due to concomitant diseases and procedure type are not well-described and deserve validated biomarkers predicting the outcomes.

In order to offer the best possible outcomes for a patient after pneumonectomy, it is necessary to identify risk factors increasing perioperative complications and early mortality. Apart from well recognized factors such as age, male gender, concomitant diseases or tobacco use there are additional factors influencing the survival. Spirometry (forced expiratory volume in 1 s, FEV1), diffusing capacity of the lung for carbon monoxide (DLCO), maximal rate of oxygen consumption (VO2max) together describe the patients’ state of respiratory and cardiovascular function. Patients scheduled for pulmonary resection should undergo assessment of their predicted postoperative values of DLCO and FEV1. If both of these values are >60% they may be accepted for surgery with low risk. If one of the predicted values is between 30% and 60% the protocols recommend the use of low tech test. If shuttle walk test (SWT) is above 400 m or stair climb test (SCT) is >22 m (height of ascent) these patients are characterized by low risk of surgical procedure. If the low-tech tests’ (SWT, SCT) results are poorer than the thresholds or the predicted postoperative values are lower than 30% these patients should undergo VO2max assessment by the means of cardio-pulmonary exercise test (CPET). If the VO2max is <10 mL/kg/min or <35% the risk of the resection is high. The VO2max value between 10 and 20 mL/kg/min or 35–75% define moderate risk of resection [6,7].

Low-tech tests are of interest due to consistently strong evidence of being good predictors of early complications as well as mortality [8,9]. Literature defines 500 m as the reliable cut-off value for assessing the risk of peri-operative complications and mortality in patients undergoing lobectomy [10]. These studies characterize the patients undergoing lobectomy. Pneumonectomy causes significant changes in the cardiovascular and respiratory system, therefore it is a more prominent trigger of complications and early mortality in vulnerable patients than lobectomy. The question is not whether the low-tech tests should replace the classic methods of pre-operative assessment (spirometry, CPET), but how they can complement them. Sole use of low-tech test for assessment of patients before resection may be tempting in patients with persistent contraindications for spirometry and CPET. Moreover, deficiencies in the availability of healthcare personnel and routine methods may force clinicians to find new, simple ways to continue care of lung cancer patients despite obstacles.

The aim of this study was to validate the established cut-off of 500 m obtained in the 6-min walking test (6MWT) in identification of patients with high and low risk of peri-operative complications, early and mid-term mortality, and overall survival after pneumonectomy performed due to non-small cell lung cancer (NSCLC).

## 2. Materials and Methods

**Figure 1:**
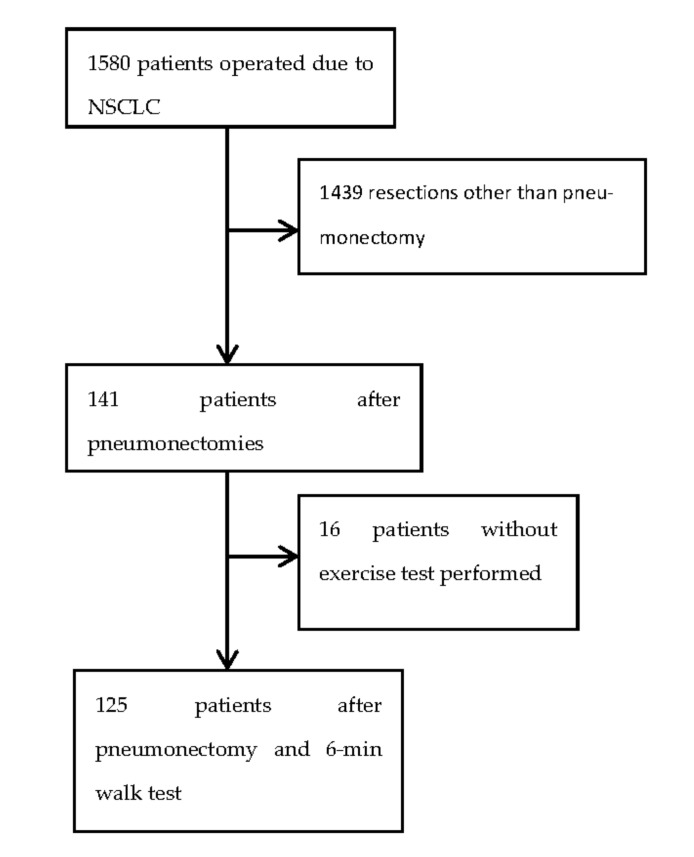
Inclusion flowchart. NSCLC non-small cell lung cancer.

A total of 1580 patients were operated in the single center due to NSCLC between January 2009 and January 2018. A homogenous group of 125 patients who performed a 6MWT one day before undergoing pneumectomy, were included in this retrospective study. The detailed study accrual is described in Figure 1. Sixteen patients who did not undergo 6MWT due to different reasons (mainly difficulty moving) were characterized in Table 1. The prospectively collected data from the Polish National Lung Cancer prospective database (Krajowa Baza Raka Pluca-KBRP) were matched with 6MWTs routinely performed in the department.

All patients underwent standard preoperative assessment of fitness for radical therapy in lung cancer (spirometry and DLCO, predicted postoperative (ppo) spirometry and ppoDLCO were assessed in patients with FEV1% < 80%). All patients with co-existing significant cardiovascular comorbidities (coronary heart disease, valve diseases, heart arrhythmias) were consulted by cardiologist. Echocardiography with left ventricle ejection fraction and pulmonary artery pressure assessments were commonly performed. Therapeutic decisions were made during a multi-disciplinary meeting. The patients not scheduled for surgical treatment due to significant coexisting morbidities were qualified for radio- or concurrent chemoradiotherapy, depending on the local advancement of the disease.

The patients were admitted one day before the scheduled operation. All of them had a diagnostic bronchoscopy performed before the surgery. Pneumonectomies were performed by postero-lateral muscle-sparing thoracotomy. In post-operative period pain was treated with epidural bupivacaine together with patient-controlled analgesia opioid pumps eventually tittered with subcutaneous morphine together with paracetamol and non-steroid anti-inflammatory drugs. Routine prophylaxis with cefazoline, low-molecular-weight heparin and proton-pump inhibitors were administered. Majority of patients were extubated in the operating room. One pleural drain 28 French was left in pleural cavity and was removed once the fluid became serous, independent of its volume. Rehabilitation was started on day zero. The patients were assisted to walking on the day one.

Complications were recorded based on the national definitions relating to KBRP. They were arbitrarily grouped to cardiovascular, pulmonary and general as listed in Table 2. Cardiopulmonary complications are the sum of cardiovascular and pulmonary complication rates.

Patients performed the 6MWT on the day before surgery. The tests were performed according to the American Thoracic Society guidelines [11]. Blood pressure was measured before and after every test. The test was performed in a 33 m-long hospital corridor. Two certified physical therapists supervised the tests. Every patient was self-paced depending on fatigue. Resuscitative backup was provided by physiotherapists regularly trained in basic life and supported by the physician with defibrillator available on the floor where the test was performed.

The reference values were counted using the previously published formula [10]. The result of the reference equation was assumed as 100% of reference value.

Despite the evidence derived from the literature we do not assume 6MWT to be an independent tool to evaluate patients before radical therapy for lung cancer. This test was used additionally to standard risk assessment protocols published by the European Society of Thoracic Surgeons [6,7]. Follow-up was assessed for every participant. Chi square test was used for categorical variables. Unpaired data characterized by normal distribution were compared with unpaired *t*-test. In case of skewed distribution, the Mann-Whitney U-test was applied. The level of statistical significance was *p* < 0.05. Odds ratios (ORs) were calculated with 95% confidence interval (CI). Receiver Operating Characteristic (ROC) curves were used to estimate the optimal thresholds (30-day, 90-day and 1st-year mortality) for a binary classifier (500 m covered in 6MWT). The curves were assessed with the Youden index method.

Follow up concerning overall survival was established for all the patients from the data available in KBRP. The median follow up was 25 months.

The study protocol was approved by the Institutional Review Board (NKBBN/88/2016).

## 3. Results

There were 93 men and 32 women with a median age of 63 years operated in the study period. All patients underwent pneumonectomy due to primary lung cancer. Detailed characteristics of patients included in the analysis are presented in Table 2.

**Figure 2:**
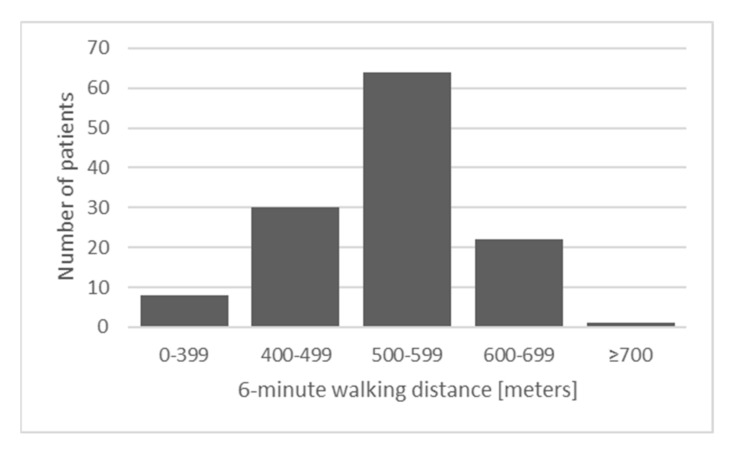
Distance covered during 6-min walking test.

The histogram presenting the distribution of distance covered during 6MWT is presented in Figure 2.

The complications are listed in Table 3. The suggested cut-off value of 500 m identified patients with increased 90-day mortality and first-year mortality, while 30-day mortality fairly reached statistical significance. Patients who covered distance shorter than 500 m had increased risk of atrial fibrillation and cardiac complications. The rates of other complications, and duration of postoperative stay did not differ between the study populations. No delayed extubations were recorded.

**Figure 3:**
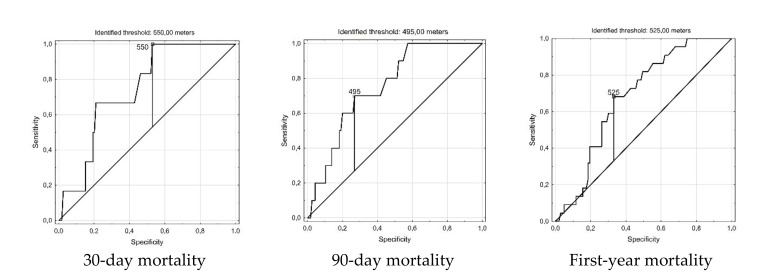
Receiver operating curves presenting different threshold values optimally differentiating worse and better early and mid-term survival.

The ROC curves method enabled evaluation of exact cut-off values of 6MWT discriminating the patients with different early and mid-term mortalities. The method identified that maximal sensitivity and best specificity was obtained for 550 m (30-day mortality; AUC 0.743, 95% CI 0.592–0.894), 495 m (90-day mortality; AUC 0.753, 95% CI 0.627–0.88) and 525 m (First-year mortality AUC 0.684; 95% CI 0.581–0.788), presented in Figure 3.

**Figure 4:**
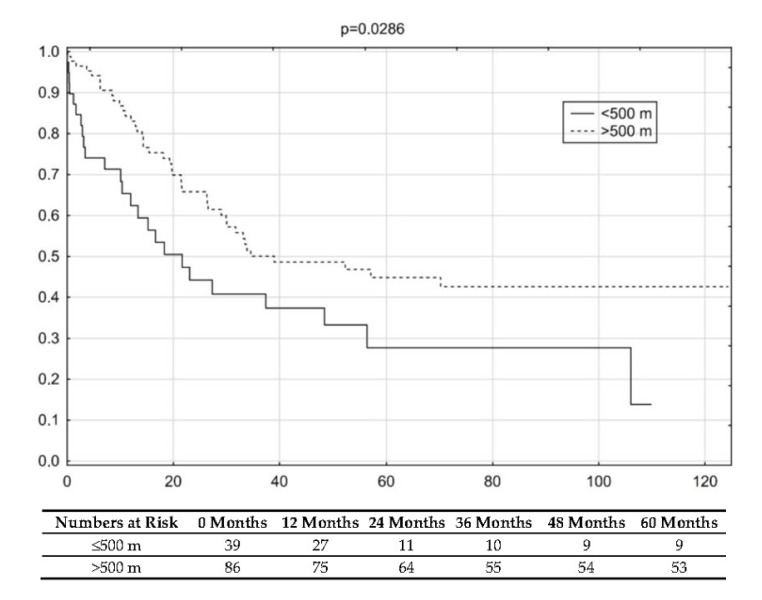
Comparison of overall survival in study groups; (HR 1.59, 95% CI 0.96–2.62).

The long-term survival differed significantly between the study groups (Figure 4). When the study population was divided based on the distance of 500 m covered in 6MWT, the difference in overall survival (OS) was observed (*p* = 0.02), (HR 1.59, 95% CI 0.96–2.62).

Univariate analysis revealed that none of the factors had influence on long-term overall survival, 30-day mortality, 90-day mortality and first year mortality. Similarly, none of the factors had influence on the presence of cardiac and pulmonary complications as presented in Table 4.

## 4. Discussion

The patients who covered shorter distance had poorer short-term and long-term overall survival. This is consistent with other studies proving that 6MWT is an adequate prognosticator of postoperative complications, early, mid-term mortality and long-term survival [8,9,10,12,13,14]. The above-mentioned studies propose different thresholds differentiating populations with different risks. The previously published thresholds identifying higher risk of postoperative complications are: 300 m [10], 400 m [14], 500 m [9]. Our study identified 500 m as an adequate result of the 6MWT identifying patients with increased risk of cardiovascular complications (OR 2.537, 95% CI 1.100–5.849) in patients with early NSCLC undergoing pneumonectomy. Performing the test on a treadmill, not according to ATS criteria, does not seem to have an impact on the clinical value of 6MWT [14]. Treadmill 6MWT shows similar threshold values differentiating patients with different risks of cardiopulmonary complications after lobectomy due to lung cancer [14]. Most of the cited studies analyze data of patients who underwent lobectomy. This homogenous population enables acquisition of prompt results. However, pneumonectomy patients deserve even more detailed analysis due to less predictable pattern of eventual complications and early mortality [15]. The assessment of the risk of complications goes beyond the evaluation of pulmonary function tests. In this group of patients synergistic tests, e.g., the cardio-pulmonary exercise test, shuttle walk test or stair climb test, are included in the guidelines and treatment algorithms [6,7].

Despite significant consequences, pneumonectomy has its place in the treatment of a well-identified patient population. Efforts should be made to define the group of patients who would benefit the most from this type of surgery. There are subpopulations of patients with lung cancer in whom pneumonectomy should not be performed, mainly due to unacceptably high perioperative mortality, as high as 26% [15]. However, most centers report perioperative mortality of less than 7% [16]. In our group of patients perioperative mortality was as low as 4.8%.

Identification of biomarkers representing unified factors is vital in patients who are unable to complete classic pre-treatment tests like CPET or spirometry. Most of the contraindications to spirometry or CPET are absolute and obvious contraindications to lung cancer surgery [17,18]. However, some of the patients (those with tracheostomy, hemoptysis, significant bronchial secretions, oral lesions or bleeding) have indelible contraindications to classic tests. In this group of patients previously complementary (i.e., stair-climb test or 6MWT) tests may play key role in accepting patients for surgery. In our study group only 11% of pneumonectomy patients were unable to complete 6MWT due different reasons. This is similar to other reports when 12–20% of patients were unable to complete exercise tests [9,19,20]. The main reason for not completing the test are significant neurological deficits, significant mental deficit and lack of consent. On the other hand, the coincidence of different reasons precluding 6MWT or spirometry is rarely observed.

Prognostic biomarkers of OS in patients with early NSCLC treated operatively are a complex of clinical features. They may be classified as related to the extent of the disease (pTNM), patient’s characteristics (age, gender, pulmonary function, quality of life) and surgery (extent of the resection, operative access, post-operative complications). Molecular factors do not play a significant role as biomarkers in an early NSCLC. It is difficult to identify one factor of key importance. The hazard ratios (HR) associated with the influence on long-term survival of individual prognostic factors vary between 1.5 (older age) through 2 (ASA ≥ 3, performance status ≥ 3, older age) to 3 and more (extent of the disease, extent of surgery) [21]. One way to optimize the value of single prognostic factors are the attempts to mathematically combine them into a formula trying to assess the overall survival [21,22]. Previous study in patients after surgical treatment of early NSCLC identified that 6MWT, is a strong prognosticator (HR 0.57) while differentiating patients with different survival [8]. It was also confirmed in our analysis including pneumonectomy patients. In present study the HR in pneumonectomy patients was 1.59. The 95% CI 0.96–2.62 included 1.0, which may be due to relatively small study group. However, both survival curves seem to present clear, separated trends. Moreover, the log-rank test revealed significant difference between survival probabilities in two study groups (*p* = 0.02). Despite seemingly inconsistent results of OS comparisons we cautiously suggest that patients with poor 6MWT result have worse OS. Current study together with the presented results allows adding 6MWT to be well documented prognostic factor in early NSCLC treated operatively.

Additionally, the COVID-19 pandemic poses a significant threat to public health systems not only by leading to deaths of vulnerable people but also by paralyzing the healthcare systems. It is difficult to predict how long will the SARS-CoV-2 virus continue affecting oncological services. Decreased access to the full-spectrum of diagnostic procedures should not result in an inadequate staging or treatment-related risk assessment. ESTS performed a survey about the changes in everyday routine during the COVID-19 pandemic. During the early months of the year 2020 all the tests used previously for preoperative workup were not available in 36% of centers [23]. We postulate that due to their simplicity and well-documented body of evidence, SWT, SCT or 6MWT may be attempted to qualify for radical surgery in patients with early lung cancer during the COVID-19 pandemic. We cannot recommend the use of these tests as sole methods of pre-operative assessment. However, we point out possibilities they offer in these unusual circumstances during the pandemic. Preliminary experiences from different centers point out the feasibility of performing the 6MWT while wearing a surgical mask, if needed.

Our analysis revealed a significant proportion (30.7%) of patients who did not survive the first year after pneumonectomy if did not reach the distance of 500 m in preoperative 6MWT. First-year mortality is not a commonly discussed mid-term outcome measure in thoracic surgery, however it may be considered. While in our study we did not identify the cause for death, it is not possible to exactly assess what is the true clinical interpretation of this endpoint after pneumonectomy. We postulate that most of the deaths within the first year post-surgery are the results of inadequate physiological assessment rather than inadequate oncological staging. We believe that it is important to discuss further methods to quantify the risk of therapy also beyond the 90-day mortality. Part of the deaths between the 90-day and the end of the first year are a result of chronic progressive processes being rapidly exaggerated by pneumonectomy (chronic obturatory pulmonary disease, pulmonary hypertension etc.). Importance of survival in first months after surgery were discussed recently [16,24].

Using univariate analysis, we did not find any factor influencing the survival in our study group. The main reason is due to the relatively small study population. This may be also due to the fact that pneumonectomy itself is one of the strongest prognosticators and may reduce the influence of other factors taken into consideration. We do not state that traditionally strong factors like pTNM, age, gender do not influence the OS in patients who undergo pneumonectomy. Similarly, we interpret that lack of influence of the 6MWT is a consequence of the study sample. The difference in OS reached the level of statistical significance (*p* < 0.05) with clinically significant HR 1.59 and 95% CI 0.96–2.62. This finding is consistent with results of other studies. Jones et al. identified the result of 450 m in 6MWT as a reliable biomarker of all-cause mortality in stage IIIB-IV NSCLC with HR 0.48 95%, CI 0.24–0.93 [25]. Corresponding threshold value of the result of 6MWT was proposed by Kasymjanova et al. Authors of this study indicated that in patients in stage IIIA-IIIB NSCLC threshold of 400 m is adequate to differentiate groups characterized by different OS HR 0.44, 95% CI 0.23–0.83 [26].

The main limitation of our study is the lack of reporting the cause for limited result of 6MWT. Detailed integrative analysis of complex cardiovascular, respiratory, and musculoskeletal function was not the subject of the analysis. Another limitation would be the lack of inclusion of patients who were not accepted for pneumonectomy due to unsatisfactory result of physiological assessment. This may lead to some selection biases. The third limitation is a relatively small study group. The group of the 125 patients did not allow to identify any prognosticators of survival in a univariate and multivariate analysis. Lack of commonly described dependencies (TNM, gender, age) suggests that small group of gathered patients did not allow identification of independent prognostic factors. This would place the 6MWT together with classic prognostic factors for early NSCLC. Another weak point of the study is the lack of assessment of the reason of death. In Poland it is not possible to retrospectively retrieve this data without violating the personal data protection laws and these data were not collected for the purposes of this analysis.

Complex physiological biomarkers are a result of combined effect of physical fitness, existing comorbidities, extent of the disease, depressed mood, and nutritional status. These factors may be represented by 6MWT (and other submaximal and exhaustive tests) [8,9,16], quality of life (EORTC QOL C-30, SF-36) [27,28,29], performance status (ECOG scale) [30] or presence of sarcopenia [31]. Further research is required to determine the interdependence of these factors. Based on current knowledge, it is easy to make a logical error in taking premise for effect and vice versa. We consecutively suggest that 6-min walking test is a good prognostic factor of complications, short-, mid-term, and long-term overall survival in patients who underwent pneumonectomy or lobectomy due to lung cancer.

## 5. Conclusions

Patients unable to reach 500 m in 6MWT are in a high risk of postoperative death after pneumonectomy, what may be a result of increased frequency of postoperative cardiac complications. Poor result of 6MWT is a predictor of worse OS.

## Figures and Tables

**Table 1:** Characteristics of patients who did not undergo the 6-min walk test.

Clinical Characteristics	Prevalence
Tobacco smoking habit	88%
Median pack-years	32
Gender	82% male
Side	82% left
Median age	63
FEV1	2.17
FEV1%	71%
FVC	3.04
FVC%	80%
30-day mortality	0%
90-day mortality	12%
First-year mortality	18%
Rate of complications	25%
Median hospital stay [days]	8

FEV1 forced expiratory volume in first second, FVC forced vital capacity.

**Table 2:** Study characteristics.

Clinical Feature	6MWD ≤ 500 M *n* = 39 Patients	6MWD >500 M *n* = 86 Patients	*p* Value
Smoking habit	36 (92%)	73 (84%)	0.250
Pack-years	34.5 ± 18.5	30.5 ± 21.3	0.128
Male gender	28 (72%)	63 (73%)	0.865
Age	64.9 ± 7.5	62.0 ± 7.0	0.080
AACCI	4.0 ± 1.6	2.9 ± 1.3	0.003
Spirometry			
FEV1	2.2 ± 0.7	2.3 ± 0.7	0.880
FEV1%	84.0% ± 20.9	80.9 ± 19.3	0.638
FVC	3.3 ± 0.9	3.0 ± 0.7	0.395
FVC%	91.7% ± 15.9	93.8 ± 21.1	0.541
6MWT result			
6MWD	427.7 ± 75.8	572.4 ± 42.9	<0.001
6MWD%	87.3 ± 21.5	107.2 ± 16.3	0.006
7th TNM			
pIA	2 (5%)	6 (7%)	1.000
pIB	5 (13%)	8 (9%)	0.551
pIIA	10 (26%)	25 (29%)	0.692
pIIB	7 (18%)	13 (15%)	0.689
pIIIA	14 (36%)	32 (37%)	0.888
pIIIIB	0	2 (2%)	0.849
pIV	1 (3%)	0	0.684
Histology			
Squamous cell carcinoma	25 (64%)	50 (58%)	0.582
Adenocarcinoma	11 (28%)	17 (20%)	0.294
Other	3 (8%)	19 (22%)	0.050

6MWD: 6-min walking distance, AACCI: Age adjusted Charlson Comorbidity Index, FEV1 forced expiratory volume in first second, FVC forced vital capacity, 7th TNM–7th revision of the TNM classification system.

**Table 3:** Complications in the study groups.

Complications	All Patients *n* = 125	≤500 *n* = 39	>500 *n* = 86	*p* Value	95% CI
Cardiovascular	32	15 (38.4%)	17 (19.8%)	0.026	2.537 1.100–5.849
Pulmonary	44	14 (35.9%)	30 (34.8%)	0.912	1.045 0.474–2.304
Cardiopulmonary	63	23 (58.9%)	40 (46.5%)	0.197	1.653 0.769–3.556
Atrial arrhytmia(as a part of cardiac complications)	28	14 (35.9%)	14 (16.3%)	0.015	2.880 1.207–6.870
30-day mortality	6 (4.8%)	4 (10.3%)	2 (2.3%)	0.055	4.800 0.840–27.418
90-day mortality	10 (8.0%)	7 (17.9%)	3 (3.5%)	0.005	6.271 1.528–25.739
1-year mortality	22 (17.6%)	12 (30.7%)	10 (11.6%)	0.009	3.378 1.310–8.709
Hospital stay		8.0	7.5	0.180	

95% CI 95% confidence interval.

**Table 4:** Univariate analysis.

Clinical Feature	30-Day Mortality	90-Day Mortality	First Year Mortality	Long-Term Survival	Cardiac Complications	Pulmonary Complications
Side (left vs. right)	*p* = 0.247	*p* = 0.596	*p* = 0.976	*p* = 0.302	*p* = 0.565	*p* = 0.122
pTNM (I + II vs. III + IV)	*p* = 0.158	*p* = 0.700	*p* = 0.216	*p* = 0.239	*p* = 0.774	*p* = 0.293
6MWT (≤500 m vs. >500 m)	-	*p* = 0.335	*p* = 0.442	*p* = 0.135	*p* = 0.370	*p* = 0.489
Gender (female vs. male)	*p* = 0.637	*p* = 0.566	*p* = 0.838	*p* = 0.239	*p* = 0.068	*p* = 0.184
Age (≤63 vs. >63)	*p* = 0.219	*p* = 0.467	*p* = 0.656	*p* = 0.289	*p* = 0.167	*p* = 0.425
AACCI(0–3 vs. >3)	*p* = 0.906	*p* = 0.863	*p* = 0.606	*p* = 0.769	*p* = 0.185	*p* = 0.286

6MWT: 6-min walk test, AACCI Age-Adjusted Charlson Comorbidity Index. pTNM–7th revision of the pathological TNM classification system.

## Data Availability

Data available on request due to restrictions eg privacy or ethical.
